# Evolutionary signatures of the erosion of sexual reproduction genes in domesticated cassava (*Manihot esculenta*)

**DOI:** 10.1093/g3journal/jkae282

**Published:** 2024-12-02

**Authors:** Evan M Long, Michelle C Stitzer, Brandon Monier, Aimee J Schulz, Maria Cinta Romay, Kelly R Robbins, Edward S Buckler

**Affiliations:** Plant Breeding and Genetics Section, School of Integrative Plant Science, Cornell University, Ithaca, NY 14853, USA; Department of Plant Sciences, University of California Davis, Davis, CA 95616, USA; Institute for Genomic Diversity, Cornell University, Ithaca, NY 14853, USA; Institute for Genomic Diversity, Cornell University, Ithaca, NY 14853, USA; Plant Breeding and Genetics Section, School of Integrative Plant Science, Cornell University, Ithaca, NY 14853, USA; Institute for Genomic Diversity, Cornell University, Ithaca, NY 14853, USA; Plant Breeding and Genetics Section, School of Integrative Plant Science, Cornell University, Ithaca, NY 14853, USA; Plant Breeding and Genetics Section, School of Integrative Plant Science, Cornell University, Ithaca, NY 14853, USA; Institute for Genomic Diversity, Cornell University, Ithaca, NY 14853, USA; United States Department of Agriculture-Agricultural Research Service, Robert W. Holley, Center for Agriculture and Health, Ithaca, NY 14853, USA

**Keywords:** cassava, clonal reproduction, sexual reproduction, deleterious mutations, evolution, Plant Genetics and Genomics

## Abstract

Centuries of clonal propagation in cassava (*Manihot esculenta*) have reduced sexual recombination, leading to the accumulation of deleterious mutations. This has resulted in both inbreeding depression affecting yield and a significant decrease in reproductive performance, creating hurdles for contemporary breeding programs. Cassava is a member of the Euphorbiaceae family, including notable species such as rubber tree (*Hevea brasiliensis*) and poinsettia (*Euphorbia pulcherrima*). Expanding upon preliminary draft genomes, we annotated 7 long-read genome assemblies and aligned a total of 52 genomes, to analyze selection across the genome and the phylogeny. Through this comparative genomic approach, we identified 48 genes under relaxed selection in cassava. Notably, we discovered an overrepresentation of floral expressed genes, especially focused at 6 pollen-related genes. Our results indicate that domestication and a transition to clonal propagation have reduced selection pressures on sexually reproductive functions in cassava leading to an accumulation of mutations in pollen-related genes. This relaxed selection and the genome-wide deleterious mutations responsible for inbreeding depression are potential targets for improving cassava breeding, where the generation of new varieties relies on recombining favorable alleles through sexual reproduction.

## Introduction

Cassava (*Manihot esculenta*) is a monoecious root crop grown in tropical regions around the world. Today, cassava is a major caloric source for over 500 million people, with a large number concentrated in sub-Saharan Africa (Parmar *et al*. 2017; Ferguson *et al*. 2019). Cassava is a woody shrub that naturally reproduces through outcrossing facilitated by separate male and female flowers. Although it is naturally perennial, it has been grown as an annual since its domestication 5–10 thousand years ago and vegetatively propagated through stem cuttings (Wang *et al*. 2014; Parmar *et al*. 2017). Centuries of selection have generated modern cassava varieties that produce large and abundant roots. This is particularly beneficial in sub-Saharan Africa where it is valued for its ability to grow with minimal inputs in marginally fertile lands, achieving an average of ∼10 tons/hectare fresh root yield (Parmar *et al*. 2017). With continually rising demands from growing populations and impending difficulties due to climate change and other environmental considerations, breeding efforts for crop improvement in cassava have garnered increasing attention.

One hurdle that impedes contemporary breeding efforts in cassava is high levels of genetic load, made visible through heavy inbreeding depression and low reproductive fitness. Several studies quantified the level of inbreeding depression in self-fertilized cassava, such that a single generation of inbreeding can decrease fresh root yield by >60% (Rojas *et al*. 2009; de Freitas *et al*. 2016). These studies likely underestimate the impact of inbreeding depression, as only measured plants that successfully grew from self-fertilized seed can be measured, missing impacts on seed germination and sexual reproduction traits. Further evidence for poor sexual reproductive ability in cassava comes from high variability in flowering time, low numbers of female flowers, high rates of flower abortion, and low seed set (Ceballos *et al*. 2004; Souza *et al*. 2020; Oluwasanya *et al*. 2021). These limitations in seed production and viability limit breeders’ abilities to make the successful crosses inherently necessary for developing new varieties and making genetic gains. Understanding genetic load can help researchers and breeders address these problems hindering the genetic improvement of cassava.

Genetic load, including that giving rise to inbreeding depression, can be measured through the accumulation of deleterious mutations throughout the genome. Cultivated cassava clones have thousands of deleterious mutations that are disproportionately maintained in a heterozygous state through clonal propagation, masking putatively recessive effects (Ramu *et al*. 2017; Long *et al*. 2023). Domestication can increase the fixation of deleterious mutations through linkage with selected loci, by “hitchhiking”, but also by reducing the effective population size through population bottlenecks, thereby weakening the ability of selection to purge negative alleles (Moyers *et al*. 2018; Bosse *et al*. 2019). However, the genomic effects of domestication may differ in clonally propagated crops. Sexually reproducing annual crops can undergo thousands of generations of recombination as selection proceeds, but clonally propagated crops show fewer recombination events (Zhou *et al*. 2017; Chen, Huang, *et al*. 2019; Chen, VanBuren, *et al*. 2019). Thus, another factor that may contribute to the accumulation of deleterious mutations is the phenomenon known as “Muller's ratchet” (Muller 1964; Felsenstein 1974). Muller's ratchet explains the negative consequences of a lack of recombination, such as in clonal or asexual populations, where deleterious mutations are unable to be purged from the population's gene pool.

It is possible to evaluate deleterious mutations across the cassava genome, by placing it in a broader evolutionary context to detect conservation at these sites. Cassava belongs to the Euphorbiaceae, or spurge, family which is a very diverse clade within Malpighiales (Kubitzki 2014). There are over 8,000 species within the family ranging from tall trees like the rubber tree, *Hevea brasiliensis*, to the ornamental poinsettia, *Euphorbia pulcherrima*. Many species are acclimated to tropical regions; however, there are also species that are succulent and adapted to drier regions such as *Euphorbia canariensis*, or Canary Island Spurge. The few common features of uniovulate Euphorbiaceae species are “latex and laticifers, pollen morphology, and ovular and seed coat characters” (Kubitzki 2014). Cassava is known to have undergone a paleopolyploidy event that is shared with *H. brasiliensis* (Pootakham *et al*. 2017).

Although both the effect of domestication on deleterious mutations in plants and animals (Kantar *et al*. 2017; Kono *et al*. 2019), and the effects of clonal or asexual reproduction leading to the fixation of deleterious mutations have been extensively explored (Muller 1964; Dwivedi *et al*. 2023), the combination of the two in domesticated, asexually reproducing crops is rare. For example, although grapes and potatoes are clonally propagated, and maintain abundant deleterious mutations in a heterozygous state, the domestication bottleneck is not as strong as annual crops (Hardigan *et al*. 2017; Zhou *et al*. 2017). In contrast, cassava experienced a severe domestication bottleneck alongside a shift to clonal propagation that has been correlated to abundant deleterious mutations and severe inbreeding depression (Pujol *et al*. 2005; Ramu *et al*. 2017). While deleterious mutations are generally negatively correlated with the recombination rate, these mutations in cassava were not correlated, likely due to the lack of recombination from clonal propagation. In this study, we aim to use evolutionary conservation to measure the impact of deleterious mutations across the cassava genome.

Using 27 recently sequenced and assembled Euphorbiaceae species (Long *et al*. 2023), we perform genetic comparisons across a total of 52 species to detect selection signatures in cassava. This deep evolutionary comparison to species within the Euphorbiaceae family allows us to measure the impact of derived mutations on cassava genes and use intraspecific variation within cultivated cassava to detect how these mutations may be shaped by selection. In this study, we use these varied measures of evolutionary time to address the possible genomic consequences of clonal propagation across the cassava genes. We demonstrate that the interplay between evolutionary conservation and accelerated evolution traces the footprint of genetic load throughout the evolutionary history of cultivated cassava, revealing the impacts of domestication and clonal propagation on gene evolution.

## Materials and methods

### Sequencing and assembly

We gathered a total of 52 related species in addition to cassava, 27 of which we sequenced and assembled, to evaluate evolutionary conservation and selection across the cassava genome. In order to maximize the amount of evolutionary time sampled; while maintaining reliable alignments to cassava, we sampled 26 species across the Euphorbiaceae family, to which cassava belongs. These species were collected from: the Germplasm Resources Information Network and contributions from many botanic gardens across the United States including the Denver Botanic Garden, the Missouri Botanic Garden, the Montgomery Botanic Garden, the National Botanic Garden, the National Tropical Botanic Garden, The New York Botanic Garden, and the US Botanic Garden.

We then extracted DNA from leaf tissue and sequenced these individuals using Illumina NovaSeq-6000. Genome sizes were estimated using k-mer spectra created using “jellyfish” (Marçais and Kingsford 2011) with a k-value of 21, in order to estimate sequence input coverage for assembly (https://bioinformatics.uconn.edu/genome-size-estimation-tutorial/). Additional short-read sequences were downloaded from the National Center for Biotechnology Information Sequence Read Archive (SRA) (https://www.ncbi.nlm.nih.gov/sra/) corresponding to 11 unspecified Euphorbiaceae taxa that were previously part of an effort to digitize a botanic garden (Liu *et al*. 2019). We then used a short-read sequence assembler MEGAHIT (Li *et al*. 2015), with modified parameters of “-m 0.2 --no-mercy --min-count 3 --k-min 31 --k-step 20” to create contig assemblies. These parameters follow recommendations for genome assemblies of complex genomes with >30X sequence coverage (https://github.com/voutcn/megahit).

We additionally obtained long-read sequences using PacBio Sequel II for 7 species among our sampled Euphorbiaceae taxa representing a diverse sample across the family. These include: *Cnidoscolus aconitifolius*, *E. pulcherrima* (poinsettia), *Excoecaria cochinchinensis*, *Garcia nutans*, *Mallotus* sp., *Mercurialis annua*, and *Reutealis trisperma*. These sequences were assembled using Hifiasm (Cheng *et al*. 2021) utilizing default settings. An additional 14 genome assemblies from other related species were downloaded from SRA (https://www.ncbi.nlm.nih.gov/sra/) and added to our assembled genomes resulting in a total of 52 species, excluding cassava (Supplementary Table 1).

Genome quality metrics were calculated to inform their usefulness in later analyses. We calculated and reported assembly size and the length of the shortest contig for which longer and equal length contigs cover at least 50% of the assembly (N50). Benchmarking Universal Single-Copy Orthologs (BUSCO) analysis was performed using the eudicot ortholog lineage database (Simão *et al*. 2015). This metric gives a rough estimate of how well the gene-space is captured by the assembled genome, while also giving a snapshot of the level of gene duplication.

### Pan-genome annotation

For the long read assemblies, we performed genome annotation using the BRAKER2 protein homology pipeline (Brůna *et al*. 2021). This BRAKER2 pipeline utilizes ProtHint (Brůna *et al*. 2020) and a protein database consisting of the Viridiplantae clade to produce de novo gene annotations.

We combined these assembled genomes with other Euphorbiaceae public assemblies of *M. esculenta*, *H. brasiliensis*, and *Ricinus communis* to create a pan-genome panel. Orthogroup and synteny analyses were performed using GENESPACE (Lovell *et al*. 2022) using the default pipeline. These orthogroups were used to define homologous genes across cassava for all analyses.

### Multiple sequence alignment

To compensate for the large variation in assembly quality, we used a limited alignment process to align fully reconstructed genes in each species. Our methodology followed a multiple sequence pipeline from https://bitbucket.org/bucklerlab/p_reelgene/src/master/ (Schulz *et al*. 2023). Cassava transcripts were aligned to each genome while tracking UTR, intron, and exon positions. Exonic regions in the target genomes that could be aligned by ≥90% of the transcript and had the highest alignment score consolidated with the query transcript. Multiple sequence alignments were created using MAFFT “--ep 0 --genafpair --maxiterate 1000” for each cassava transcript. While this methodology ignores duplicated copies of genes in target genomes, it simplifies analyses by avoiding errors introduced from fragmented assemblies and polyploidy. Additionally, to enable in-frame protein coding analysis across homologous genes, any positions with gaps in the cassava transcript were removed from the multiple sequence alignment.

### Gene tree analysis

We performed phylogenetic analyses to assess gene evolution and selection signatures. First, we generated maximum likelihood trees using RAxML “-m GTRGAMMA -p 12345” (Stamatakis 2014) for every transcript with a minimum of 4 aligned genomes. To estimate the neutral evolutionary tree of these species, we randomly sampled 1,000 genes and concatenated the 4-fold degenerate sites (sites where mutations produce no amino acid changes) from their multiple sequence alignments. We rooted this tree to the Malpighiales species *Hypericum perforatum*. This species tree was used only for the visualization of relationships.

Next, we used the phylogenetic analysis by maximum likelihood (PAML) suite of tools to evaluate gene and site level conservation (Yang 2007). For protein coding analysis, we executed 2 different models of the PAML codeml tool. The models differ by either treating the tree as having one ω ratio parameter for the entire tree or allowing 2 ω parameters: one for the cassava branch and one for the rest of the tree. For each test, we used a gene tree generated from the gene of interest. Each model reports the ratio of nonsynonymous to synonymous variants (dN/dS) at each gene as well as the likelihood of the given model. To aid in the interpretability of dN/dS ≫ 1, we set the maximum dN/dS = 2, thresholding all values greater than this to 2. We interpreted the difference between these models as a method of detecting a difference in selection between cassava and the rest of the tree and performed likelihood ratio tests to verify the significance of any differences between these models. Additionally, we performed PAML baseml analysis for each transcript multiple sequence alignment giving an evolutionary rate for every base-pair position. For the subset of pollen-related genes found to be under significant relaxed or positive selection, we analyzed protein evolutions using a PAML Branch-Site model. This model is used to identify which amino acid substitutions are responsible for the differences between our target branch, cassava, and all the other species.

### Gene model selection

We used our evolutionary information to filter down the ∼64k and ∼33k transcript and gene from the CassavaV7.1 annotations, respectively, to 26k gene models with a single most conserved transcript. First, we filtered out any transcripts that had no annotated untranslated regions, as this suggests it has minimal RNA evidence. Next, we retained gene models that were found in at least one of the other 52 assembled species, as de novo gene generation without any homology at this timescale is likely rare. The best transcript for the remaining genes was decided by looking at the lower dN/dS ratio indicating the most evolutionary conservation and potential for functionality. Any remaining multiple transcripts at a gene were filtered to the longest transcript, which, while not necessarily ideal, provides us with a single transcript for future analysis for each of the ∼26k genes.

### Intraspecific selection signatures

We used a large panel of cassava clones to assess intraspecific measures of selection. From the haplotype map found on cassavabase.org (Fernandez-Pozo *et al*. 2015), we filtered down to 330 cassava clones with at least 10X average genome coverage. We then filtered variant sites to biallelic single nucleotide polymorphisms (SNPs) with <20% missingness and minor alleles having at least 3 occurrences. We calculated the Residual Variation Intolerance Score (RVIS; Petrovski *et al*. 2013), by regressing the number of nonsynonymous SNPs with a minor allele frequency ≥1% onto the total number of SNPs in each gene, then scoring each gene's studentized residual from this regression. To further focus on those functional variations likely to represent deleterious load, we performed the same analysis on nonsynonymous sites flagged as putatively deleterious. These deleterious mutations were classified previously (Long *et al*. 2023) and are sites with a baseml evolutionary rate <0.5, a Sorting Intolerant from Tolerant (SIFT) (Ng and Henikoff 2003) score ≤0.05, and a minor allele frequency <20%. The RVIS methodology was then repeated to form a score we term here as deleterious RVIS (DRVIS).

We also combined interspecific and intraspecific measures of functional variations to test for positive selection. The test performed was the “McDonald–Kreitman test” (McDonald and Kreitman 1991) which can be calculated as α = 1 − (pNpS/dN/dS), where pNpS is the ratio of nonsynonymous mutation sites to synonymous mutation sites within a population. We used the cassava hapMap (Ramu *et al*. 2017) population to determine pNpS, using a minor allele frequency cutoff of 10%, and calculated α. Similar enrichment tests to those previously described were performed with α, where the α > 0 describes the proportion of sites fixed by positive selection (Supplementary Table 3 and Fig. 3).

### Selection evaluation

We collected gene ontologies (GOs) through homology to the TAIR10 *Arabidopsis thaliana* genes (https://www.arabidopsis.org/). BLASTP was performed between CassavaV7 and TAIR10 to determine homologous genes. GO term enrichment was performed using the “topGO” package in R (Version 2.50.0), analyzing GO terms for biological processes in regard to each of our selection measures (Tables 1–3, Supplementary Table 3). GO terms were filtered to only include terms that are present in more than 1 gene. GO term significance is the result of a Fisher exact test with the associated *P*-value. The top 10 GO terms for each test were reported, and with a total of 5,828 GO terms tests, a Bonferroni multiple test correction *P*-value threshold of 8.57e−6 can be used to consider significance. We used public databases of *A. thaliana* gene expression atlases (Waese *et al*. 2017, https://bar.utoronto.ca/eplant/) to compare tissue-specific expression of orthologous genes to the set of 48 genes with relaxed selection signatures (Supplementary Table 2; Nakabayashi *et al*. 2005; Schmid *et al*. 2005).

**Table 1.** Enriched GO terms for genes under relaxed selection.

GO.ID	Term	Annotated	Significant	Expected	*P*-value
GO:0009860	**Pollen tube growth**	177	6	0.34	5.1e−06
GO:0060321	**Acceptance of pollen**	12	2	0.02	0.00023
GO:0006893	Golgi to plasma membrane transport	27	2	0.05	0.00122
GO:0009409	Response to cold	652	6	1.25	0.00143
GO:0072583	Clathrin-dependent endocytosis	46	2	0.09	0.00350
GO:0009651	Response to salt stress	940	7	1.80	0.00495
GO:0006900	Vesicle budding from membrane	64	2	0.12	0.00667
GO:0009846	**Pollen germination**	79	2	0.15	0.01001
GO:0046777	Protein autophosphorylation	264	3	0.50	0.01397
GO:0010224	Response to UV-B	108	2	0.21	0.01814

GO term enrichment produced from “topGO” among genes significantly relaxed from selection (ΔdN/dS < 0 and Bonferroni multiple test correction significance). Pollen related GO terms are shown in bold.

**Table 2.** Top 10 enriched GO terms for genes with excessive nonsynonymous mutations (RVIS genes).

GO.ID	Term	Annotated	Significant	Expected	*P*-value
GO:0006952	Defense response	3,176	250	138.86	6.0e−15
GO:0007165	Signal transduction	3,162	186	138.24	1.2e−10
GO:0006468	Protein phosphorylation	1,473	118	64.40	1.7e−09
GO:1902065	Response to L-glutamate	19	9	0.83	3.5e−08
GO:0042742	Defense response to bacterium	1,308	101	57.19	2.7e−07
GO:0007166	Cell surface receptor signaling pathway	71	15	3.10	3.4e−07
GO:0050832	Defense response to fungus	831	64	36.33	6.9e−06
GO:0010483	**Pollen tube reception**	26	8	1.14	1.0e−05
GO:0032922	Circadian regulation of gene expression	35	9	1.53	1.4e−05
GO:0009616	RNAi-mediated antiviral immune response	28	8	1.22	1.8e−05

GO term enrichment produced from “topGO” among genes in the top 5% of RVIS scores. Pollen related GO terms are shown in bold.

**Table 3.** Top 10 enriched GO terms for genes with a buildup of deleterious mutations (DRVIS genes).

GO.ID	Term	Annotated	Significant	Expected	*P*-value
GO:0006468	Protein phosphorylation	1,473	144	75.04	2.6e−19
GO:0080168	Abscisic acid transport	24	13	1.22	2.1e−11
GO:0048544	**Recognition of pollen**	100	25	5.09	2.2e−11
GO:0090332	Stomatal closure	99	17	5.04	1.9e−06
GO:0010496	Intercellular transport	43	9	2.19	3.2e−06
GO:0015692	Lead ion transport	19	7	0.97	2.5e−05
GO:0019318	Hexose metabolic process	114	13	5.81	3.6e−05
GO:0007165	Signal transduction	3,162	197	161.09	4.8e−05
GO:0090436	Leaf pavement cell development	10	5	0.51	6.9e−05
GO:0051645	Golgi localization	10	5	0.51	6.9e−05

GO term enrichment produced from “topGO” among genes in the top 5% of DRVIS scores. Pollen related GO terms are shown in bold.

Cassava underwent a paleopolyploidy event and many genes contain duplicates throughout its genome. Because of this genome duplication, we addressed our measures of selection on individual genes as well as consolidating their metrics by ortholog group, by recording the least extreme value (i.e. smallest absolute value or least significant *P*-value). Additionally, we analyzed differences in selection signatures between paralogues within ortholog groups (Supplementary Fig. 6) and measured evolutionary distances to each assembled species and known homeologous chromosome pairs (Bredeson *et al*. 2016) to determine if there is any evidence for allopolyploidization, which would show asymmetric similarity to a relative of a putative diploid progenitor (Supplementary Fig. 4). Additionally, we binned our selection measurements (dN/dS, RVIS, DRVIS, etc.) and other genome annotations (gene density, genetic map, and domestication sweeps; Ramu *et al*. 2017) into 250 kb bins to examine large-scale signatures of asymmetric selection across homeologous chromosomes (Supplementary Fig. 5).

### Differential expression

Differential expression between flower (mixed female and male inflorescences) and nonflower tissues was performed for the 48 genes found to be under relaxed selection. RNA sequence counts across 5 different tissues and 150 cassava clones from a previous study were used to evaluate gene expression (Ogbonna *et al*. 2021). Differential expression analysis was performed using R package “DEseq2” (Love *et al*. 2014), comparing flower tissue expression to all other tissues. This analysis compares the 2 sets of expression data, flower tissue and nonflower tissue, and reports the Log2Fold changes in expression and the associated *P*-value for each gene. This was used to help support the floral function of the annotated genes. Log2Fold changes in expression with significance levels were reported (Supplementary Fig. 1).

## Results

### Genome assemblies

In conjunction with a breeding study, we sequenced and assembled 27 genomes for the purpose of training a machine-learning model to improve genome-wide selection models for breeding (Long *et al*. 2023). In this study, we annotate and characterize these genomes to compare ortholog conservation across the cassava genome. Most of these species were sequenced using short-reads, while 7 species were sequenced using long-read sequencing. In addition to these sampled species, we assembled 11 Euphorbiaceae taxa with publicly available short-read sequences from a botanic garden survey (Liu *et al*. 2019), and collected 15 public reference assemblies (Tuskan *et al*. 2006; Bredeson *et al*. 2016; Xu *et al*. 2017; Horvath *et al*. 2018; Chen, Huang, *et al*. 2019; Chen, VanBuren, *et al*. 2019; Jalali *et al*. 2020; Liu *et al*. 2020; Wei *et al*. 2020; Wu *et al*. 2020; Zhou *et al*. 2020; Cai *et al*. 2021; He *et al*. 2021; Zhou *et al*. 2021; Lu *et al*. 2022) for a total of 53 species (Supplementary Table 1). Genome sizes and sequence coverage were estimated through k-mer analysis (Supplementary Table 1).

**Fig. 1.**
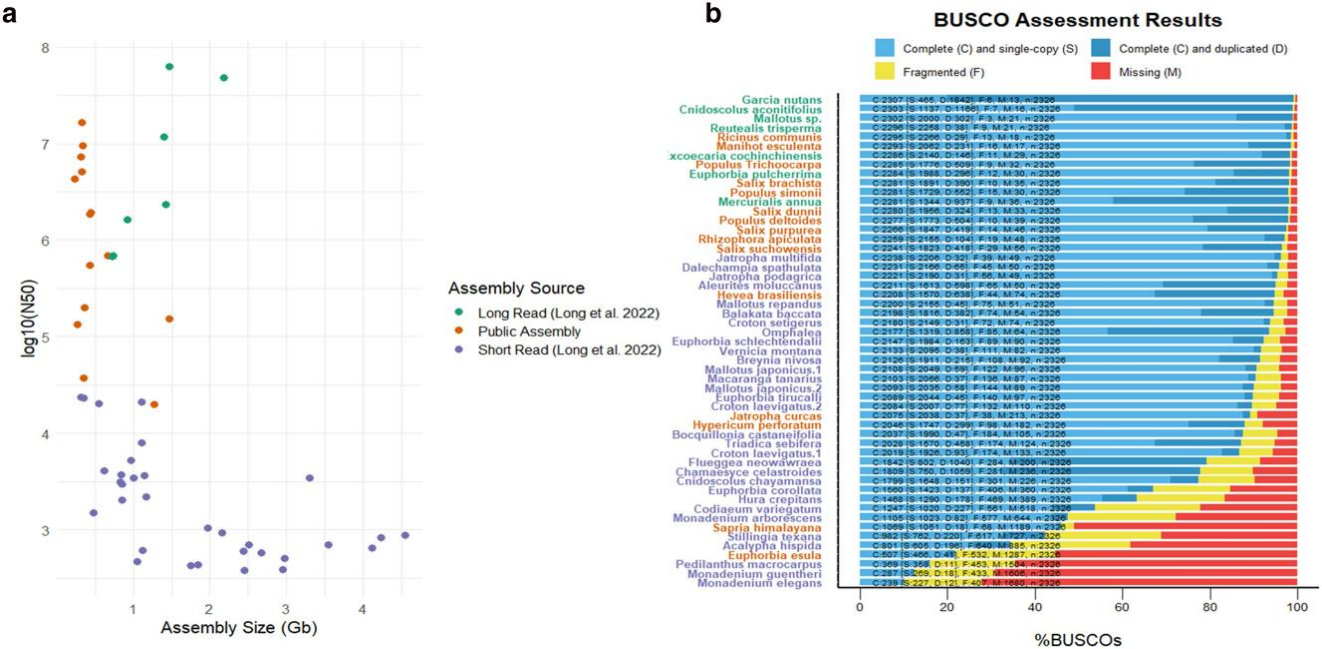
Assembly quality statistics. Assembly size and N50 are shown for long and short-read assemblies we produced, as well as the public assemblies used in this study (a). BUSCO scores are plotted with species’ text color matching sources (b).

Genome assembly quality was evaluated by assembly size, contiguity, and reconstructed gene-space (Fig. 1). The quality of gene-space reconstruction was estimated through BUSCO, and contiguity quality represented by the length of the contig of which 50% of the assembly is contained with that size of contig or larger (N50). These assembled genomes have large variability in contiguity and quality of gene-space reconstruction due to differences in sequencing methods as well as large variability in genome size (Fig. 1). Species assembled from long-reads are of very high quality with N50 and BUSCO values comparable or higher to many of the previously published reference assemblies.

### Pan-genome annotations

We combined our long-read assemblies with publicly available Euphorbiaceae genome assemblies to create a Euphorbiaceae gene pan-genome. Our de novo assemblies were annotated using BRAKER2, and genome homology and synteny were produced through the tool GENESPACE (Lovell *et al*. 2022). This pan-genome defines orthogroups for each gene, including cassava genes. There are over 11k orthogroups that are found in at least 80% of high-quality Euphorbiaceae assemblies in our pan-genome (Supplementary Fig. 1). These conserved orthogroups account for ∼19k cassava genes (72% of the high-quality genes) and are likely biased toward syntenic genes.

### Evolutionary conservation across the Euphorbiaceae family

**Fig. 2.**
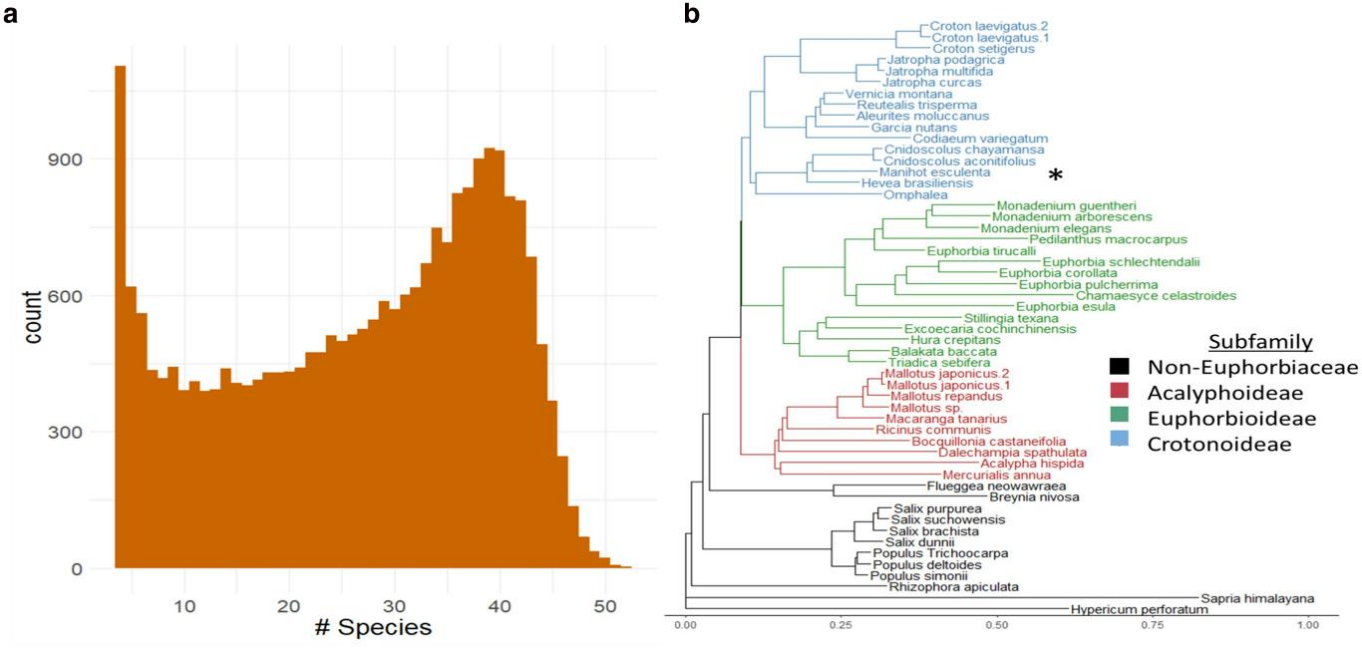
Ortholog occurrence across all assemblies and phylogenetic relationships. An ortholog frequency histogram with the number of species that are represented in each ortholog group across all assemblies (a). Phylogenetic tree created from 4-fold degenerate sites from 1,000 randomly selected genes. The Euphorbiaceae subfamilies are designated by color (b). Cassava (*M. esculenta*) is part of the Crotonoideae subfamily and designated with “*”.

We measured the presence of reconstructed gene orthologs across our phylogeny and found a wide distribution of how many genome assemblies had complete homologous sequence for each cassava gene (Fig. 2a). We considered alignments for the single best-aligned homologous gene from each genome assembly with at least a 90% aligned length to the cassava homolog. While the distribution of taxa with homologous genes is dependent on assembly quality, there are many genes that are present and completely assembled across a majority of Euphorbiaceae and related species, even from the short-read assemblies. We see very few genes across all 53 species, likely hindered by poor assembly and alignment quality derived from short-read assemblies. The set of cassava genes with very few observations across the Euphorbiaceae may indicate those genes are either unique to cassava, conserved in few species, or may be nonfunctional annotations thus not conserved.

We constructed a phylogeny to evaluate relationships among these 53 species. A maximum likelihood neutral tree was estimated from 4-fold degenerate sites in a random sample of 1,000 genes (Fig. 2b). This phylogeny shows relationships that agree with previously understood taxonomic relationships, including 3 subfamilies: Acalyphoideae, Crotonoideae, and Euphorbioideae (Wurdack *et al*. 2005). Two species, *Breynia nivosa* and *Flueggea neowawraea*, were previously classified as biovulate subfamily Phyllanthoideae, and are now part of a separate family Phyllanthaceae (Wurdack *et al*. 2005). Outgroup species, including aspen, willow, and flax, are distantly related but still fall within the order of Malpighiales.

While the contiguity and quality of short-read assemblies are relatively low (Fig. 1a), their assembly of genic regions allowed us to incorporate these species into evolutionary assessment. Many of the short-read assemblies from genomes that were ≤1 Gbp in size had high contiguity and BUSCO scores, while those from species with larger genome sizes are of lower quality. This is mainly due to common sources of difficulty such as obtaining high enough coverage sequence data and assembling large complex regions with short-read information. Ultimately the utility of these genomes is visible in the amount of reconstructed ortholog space in the cassava genome (Fig. 2a).

### Interspecific selection signatures

**Fig. 3.**
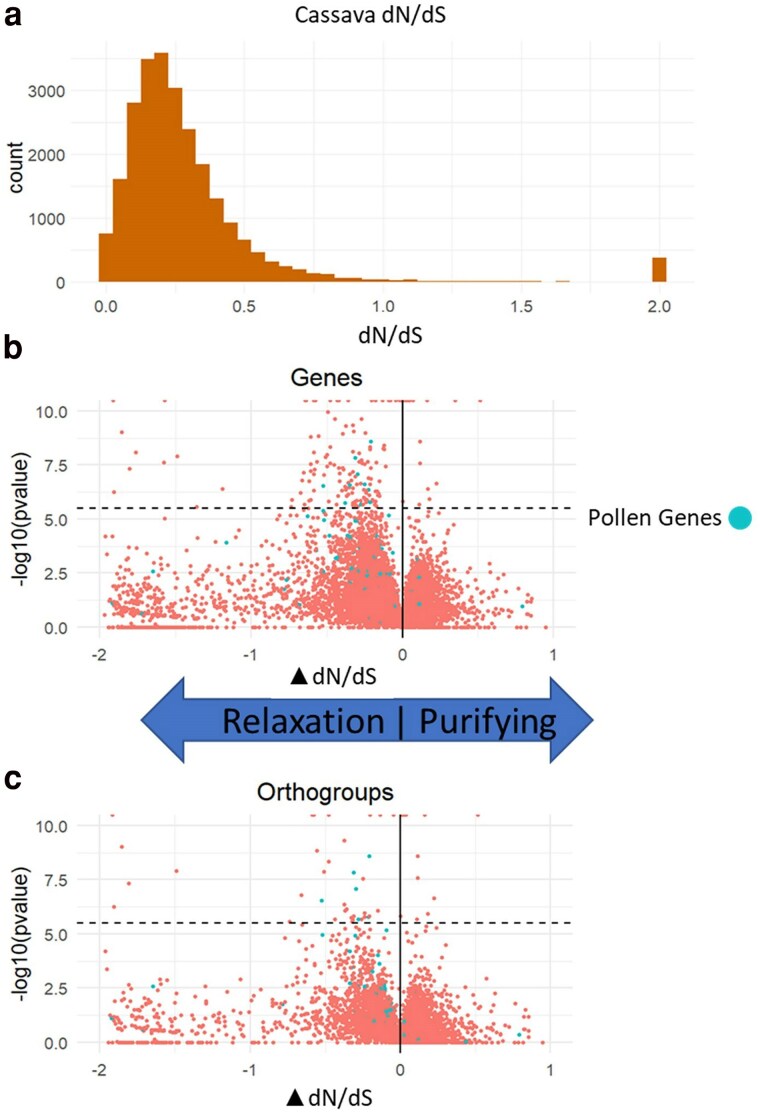
Selection signatures dN/dS gene conservation. a) Histogram of dN/dS values from all genes across cassava, with values >2 plotted at dN/dS = 2 (top). b) The difference in dN/dS score between the 52 species used in this study and cassava for each gene in cassava, with the *y*-axis showing the log ratio test *P*-value between these 2 models and dotted line showing multiple test correction significance threshold. c) The difference in dN/dS score between the 52 species used in this study and cassava summarized for each orthogroup in cassava, with the *y*-axis showing the log ratio test *P*-value between these 2 models and dotted line showing multiple test correction significance threshold. Arrows indicate difference in selection in cassava (i.e. ΔdN/dS < 0 implies a relaxation of purifying selection in cassava, or a transition to more positive selection, and ΔdN/dS > 0 implies a stronger purifying selection in cassava).

Using evolutionary conservation and interspecific variation, we analyzed selection signatures across ∼26k cassava gene models that passed quality filters. We calculated the ratio of nonsynonymous, causing amino acid changes in protein sequence, to synonymous, putatively neutral, substitutions across the evolutionary tree (dN/dS, Kryazhimskiy *et al*. 2008, Fig. 3a). Additionally, we estimated this ratio for substitutions occurring along the cassava branch of the tree. Comparing nucleotide sequence conservation across millions of years allowed us to measure the selection on cassava genes. Our results show the majority of genes in the cassava genome have a dN/dS < 0.5 implying purifying selection (Fig. 3a). These genes are likely functional and important to be conserved across the Euphorbiaceae family and related species, and to have low tolerance for mutations that disrupt conserved function. Genes with dN/dS ∼ 1 are likely not under strong selection and are either nonfunctional or whose function is not currently beneficial. Genes with dN/dS > 1 are either under positive selection, relaxed purifying selection, pseudogenes, or are poorly estimated due to short gene length (Fig. 3a). For visual clarity we truncated all dN/dS ratios >2, setting their value to 2. Using the PAML branch test, we tested the relaxation of selection in cassava and reported the difference between the dN/dS of the evolutionary tree and that of the cassava branch. The negative ΔdN/dS indicates a larger dN/dS value in the cassava branch of the tree, or a transition away from purifying selection. By comparing this branch-specific ratio to the value estimated across all species we found 167 genes comprising 148 different orthogroups that showed significantly higher dN/dS values along the cassava lineage (Supplementary Table 2), suggesting relaxed or positive selection in cassava (Fig. 3b). We found that the reliability of these estimates of dN/dS and the significance of separations between cassava and the other species is dependent on the length of the coding sequence and the number of species with reconstructed orthologs, with several short genes experiencing large, but insignificant differences in dN/dS ratios (Fig. 3b and c—lower left quadrants). This results from limited observations of the ratio of nonsynonymous to synonymous mutations that can result in extremes that are not statistically relevant.

The ancestor of cassava experienced a whole genome duplication ∼40 million years ago (Bredeson *et al*. 2016), and genes resistant to fractionation have been retained as duplicated genes across the genome. Duplicate genes may show signatures of relaxed selection, if one copy maintains function and the other subfunctionalizes or neofunctionalizes (Flagel and Wendel 2009). To minimize the possibility of conflating ongoing fractionation with relaxed selection on ancestral function, we only considered orthologous groups of genes that contained either a single cassava gene or where all cassava gene copies passed significance tests, resulting in 48 genes from 47 orthogroups (Fig. 3c).

Since its domestication, selection in cassava has been strongest on root mass. We expected traits unnecessary for the clonal reproduction of these large roots such as photosensitivity, flowering and seed production, and perennialism to be released from selection. We performed differential gene expression using available RNA-sequencing data from 5 cassava tissues and found that from among the 48 genes showing relaxed selection, 15 had differentially increased expression in flowers compared to nonflower tissues (Supplementary Fig. 2). Additionally, ∼70% of the *A. thaliana* homologs to the 48 genes are most highly expressed in flower, seed, and pollen tissues, (Supplementary Table 2).

We then investigated the possible biological functions of this set of genes that show relaxation from purifying selection in cassava compared to the rest of the evolutionary tree. We performed GO enrichment for GO terms regarding biological processes. The set of 48 genes with significant differences in dN/dS values showed an enrichment for processes involved with pollen and pollen tube development, which exhibited a 20-fold enrichment (Table 1). These ontologies were attributed to 6 specific genes in cassava: Manes.02G178800, Manes.03G204900, Manes.03G130950, Manes.04G017000, Manes.04G056400, and Manes.08G062900, all were expressed in floral tissues and 4 were expressed differentially higher in flowers relative to leaf, stem, fibrous, and storage root tissues. While less pronounced other showed significant enrichment in the set of relaxed genes including responses to multiple stresses (cold, salt, UV-B). These may be the result of domestication and the removal of stresses that exist in the species’ wild, perennial state.

### Intraspecific selection signatures

**Fig. 4.**
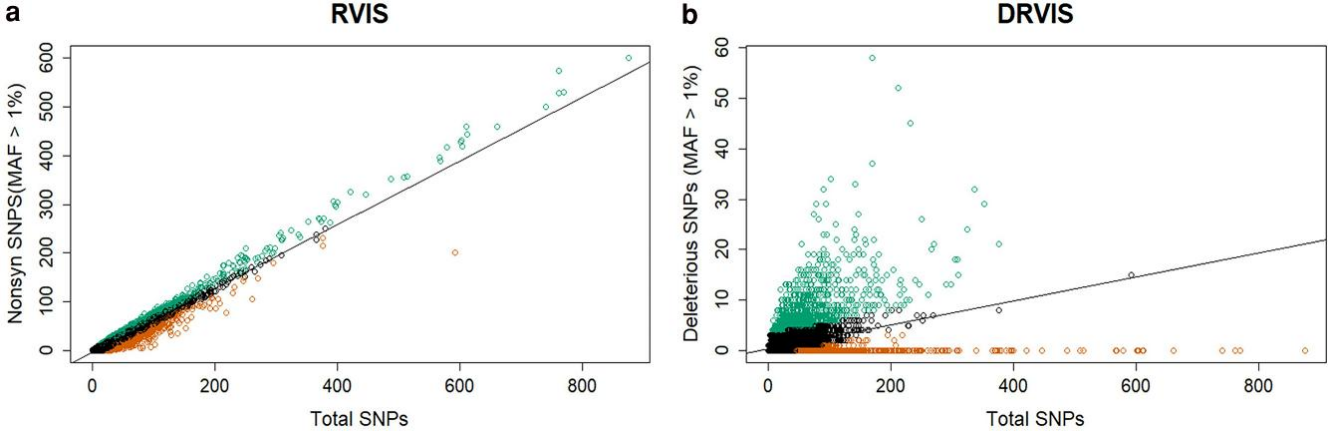
Identification of deleterious mutations in genes using within species residual variation intolerance scores. Regression for number of nonsynonymous SNPs (a) and putative deleterious SNPs (b) against the total number of SNPs in each gene in cassava. The residuals from each regression give RVIS and DRVIS scores, with the top and bottom 5% (*n* = 1287) shown.

Relaxed selection along the cassava lineage identified candidate genes, but it is unclear whether the relaxation of selection occurred across the genus *Manihot*, or after domestication. To determine which pathways were released from selection in domesticated cassava, we used intraspecific data with a sample of 330 sequenced cassava clones. We used 2 versions of the residual variation intolerance score (RVIS, Petrovski *et al*. 2013), which uses either excess nonsynonymous (Fig. 4a) or deleterious mutations (Fig. 4b) to identify outlier genes. RVIS statistics help control for protein length and differential mutation and drift at the gene level. To identify genes with extremes of deleterious variation, we calculated a DRVIS score using variant sites classified as deleterious through evolutionary conservation (DRVIS, Fig. 4b). The genes in the top 5% (*n* = 1287) of RVIS (Table 2) and DRVIS (Table 3) scores, representing those genes with high polymorphism at functional sites, also showed enrichment for pollen-related biological processes, among other biological processes including multiple processes responsible for defense against biotic stresses.

**Fig. 5.**
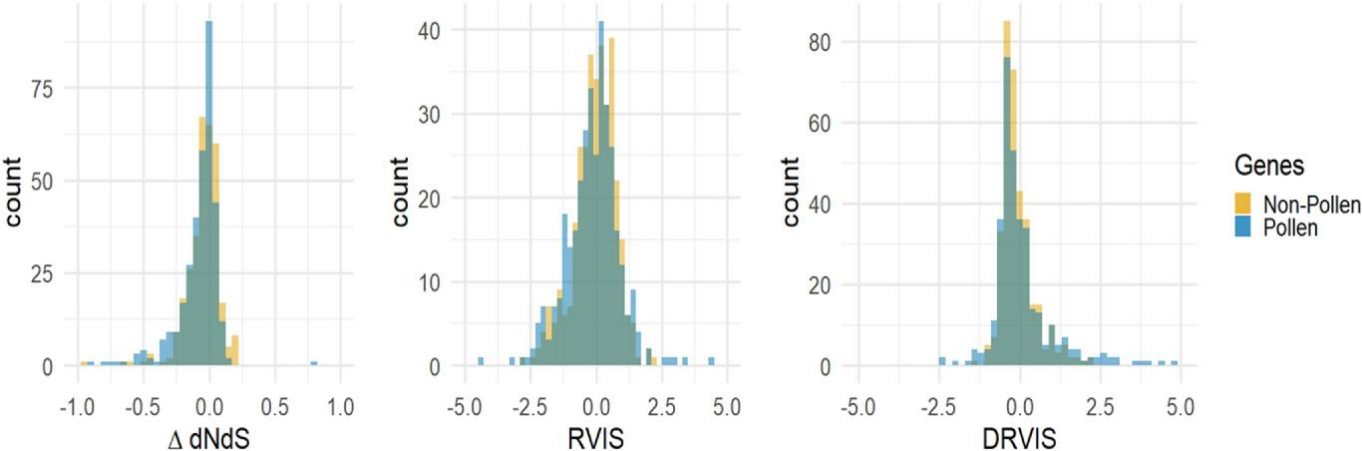
Distributions of ΔdN/dS, RVIS, and DRVIS between pollen and nonpollen-related genes. Histograms are shown between pollen (blue) and nonpollen (orange) related genes for ΔdN/dS, RVIS, and DRVIS. Nonpollen-related genes are subsampled to an equal number of genes for visual comparison.

Given that pollen-related traits are enriched among the extremes of both interspecific and intraspecific measures of genetic load, we further investigated all 348 genes that had pollen-related GO terms, irrespective of whether they reached significance in individual tests. We performed χ^2^ tests for significant differences in distributions of ΔdN/dS, RVIS, and DRVIS between 348 pollen-related genes and all other cassava genes (Fig. 5). We found ΔdN/dS (*P*-value = 0.027) and RVIS (*P*-value = 0.0055) to be significantly lower in pollen-related genes than all other genes, while DRVIS (*P*-value = 3.3e−05) was significantly higher. All these effects, while significant, are small, but are consistent with relaxation from selection among pollen genes in cassava.

In addition to dN/dS, RVIS, and DRVIS we also used the MK test to evaluate selection measures. The MK test uses a combination of intraspecific and interspecific functional variation to measure selection with a positive value (α > 0) indicating the proportion of substitutions fixed by positive selection. GO term enrichment was performed on genes in the top 5% of the MK test α value (α = 1), and found many functions related to plant defense, like RVIS (Supplementary Table 3). The MK test α value also showed enrichment for pollen tube functions, though overall no significant difference across all pollen genes (Supplementary Fig. 2). Negative estimates of α are common in plant populations (Gossmann *et al*. 2010), as we observe for the majority of genes in cassava. This may be due to low effective population sizes in cassava, intensified by the population contractions seen during the domestication and improvement bottlenecks (Ramu *et al*. 2017), altering the landscape of segregating variation. Additionally, MK tests are also sensitive to segregating slightly deleterious variation (Messer and Petrov 2013), limiting the ability to detect positive selection.

### Chromosome evolution

Knowing that the cassava genome has experienced a paleotetraploidy event, we examined previously characterized homeologous chromosomes to look for any asymmetrical measures of conservation and selection. We took the average genetic distance to each other genome assembly across all cassava genes. These distances were then compared across homeologous chromosomes to look for any evidence of chromosome lineage resulting from an allopolyploid event (Supplementary Fig. 4). We found that with the current resolution provided from the genome assemblies in this study, there is no evidence for allopolyploidization; however, this cannot be certain from the available information.

We also examined our selection metrics across the cassava chromosomes to look for any signatures of biased fractionation, due to one homeologous chromosome being selectively conserved over the other (Supplementary Fig. 5). While regions of some chromosomes show some signatures of decay (low gene densities, low recombination rates, high dN/dS values), there does not appear to be any evidence for whole chromosome degradation. However, among genes with multiple paralogues, there is evidence for gene subfunctionalization or degradation as seen by one gene copy showing high conservation over other gene copies (Supplementary Fig. 6).

## Discussion

The intricate interplay between natural selection, domestication, and prolonged clonal propagation has shaped the cassava genome over the course of its cultivated history. We explore the impact of clonal propagation after domestication, and the absence of recombination, has had on the accumulation of deleterious load in the cassava genome. Employing interspecific and intraspecific analyses, we evaluate selection signatures across the genome, corroborate with tissue-specific expression, and investigate affected gene pathways, and finding evidence for relaxed selection in pollen and flower-related genes.

### Selection signals from comparative genomics

Surveying millions of years of evolution by sampling taxa across the Euphorbiaceae, we measured selection on each cassava gene. Most genes under differential selection are relaxed along the cassava lineage (Fig. 3b and c) and are enriched for pollen-related functions. Sexual-related genes have been observed to experience faster evolutionary rates in general (Cutter and Ward 2005), and these genes may be predisposed for faster mutation accumulation. Asexual reproduction may allow for the accumulation of increased mutational load in sexual function genes, as these genes are likely no longer required for effective reproduction. Related breakdowns in genes involved in pollen function have been observed between outcrossing wild species and clonally propagated cultivars, for example in potato (Hardigan *et al*. 2017) and the ornamental *Ranunculus* genus (Kocot *et al*. 2022). Even wild species may show signatures of this process. In the tree species *Populus tremuloides*, male fertility declines with the clonal age of an individual, potentially due to the accumulation of somatic mutations (Ally *et al*. 2010). In *Decadon verticillatus*, a transition to asexual reproduction led to the loss of sexual compatibility, primarily through pollen dysfunction (Eckert *et al*. 1999).

Comparative evolutionary signal supports a role in the sexual reproduction of the 48 relaxed cassava genes. Two of these genes, Manes.02G178800 and Manes.08G062900 are annotated as exocyst and secretory complexes that have been experimentally shown to be necessary for proper pollen development in *A. thaliana* (Marković *et al*. 2020). Two more genes, Manes.03G130950 and Manes.04G017000, are annotated as members of the ATP-binding cassette transporter family, whose homologs are essential for anther and pollen exine development in rice (Qin *et al*. 2013).

### Intraspecific selection signals

Genes under relaxed selection in the reference genome of *M. esculenta* relative to other taxa could represent episodes of selection that happened during speciation, or during domestication. To disentangle these effects, we used genotyped cassava clones to provide a within species perspective on selection. Similar to the evolutionary signal seen in comparative genomics across the Euphorbiaceae, genes with pollen-related functions showed enrichment for functional variation, measured by RVIS and DRVIS, between cassava clones. Further analysis of RNA expression data in cassava supports the sexual reproduction-related functions of the 48 genes that showed relaxed selection, with many of them showing differentially increased expression in flower tissues (Supplementary Fig. 2).

While GO term enrichment may be prone to overinterpretation, it is a common method used to identify possible affected biological processes and combined with other analyses can be one signal supporting a conclusion. Other significantly enriched GO terms from these population-level analyses included defense response functions. This may result from disruptive positive selection, as high amounts of functional variation can be beneficial. Selection for the high diversity of plant defense genes has been previously shown in plants (Zhang *et al*. 2014, 2016), and these defense response genes may be relevant for common diseases afflicting cultivated cassava such as cassava mosaic virus or cassava brown streak disease. Genes with functions related to circadian rhythms also showed evidence for positive selection, which agrees with previous understanding of gene functions in plants commonly under positive selection (Michael *et al*. 2003). None of our analyses resulted in pathways involved in root development or function, even though large amounts of selection have been applied to this trait. This may be because GO terms derived primarily from studies of Arabidopsis may not have a direct correlation to pathways responsible for the development of large storage roots like those found in cassava. While GO terms are a coarse estimation for function, these and the other significant functional elements may be further investigated to shed light on molecular functions behind cassava evolution, domestication, and cultivation.

Sexual recombination and seed production are essential to combine favorable alleles to create improved cultivated varieties. Low fruit and seed production hinders breeding efforts in cassava. Low rates of female flower production and large variation in flowering times have been targets to alter for increased cassava seed production (Oluwasanya *et al*. 2021). Flowering induction is only part of the problem, however, as many studies have reported flower abortion rates of over 80% (Ukwu *et al*. 2018; Ramosy Abril *et al*. 2019; Bandeira E Sousa *et al*. 2021). Studies have also found genotypic variability in pollen viability in cassava, and that self-incompatibility does not explain this variability (Ramos Abril *et al*. 2019; Bandeira E Sousa *et al*. 2021). It has been suggested that fruit abortion in outcrossing species may be due to deleterious mutations (Wiens *et al*. 1987). Multiple studies have shown low pollen amounts and low pollination rates in cultivated cassava crosses compared to wild progenitors and other Manihot species (da Jennings 1963; Vieira *et al*. 2012; Silva *et al*. 2018). Previous studies on the relationship between cassava clonality on deleterious mutations have shown an unexpected lack of correlation between recombination and deleterious mutations (Ramu *et al*. 2017), supporting the conclusion that these mutations are indeed being enriched through absent recombination from clonal reproduction.

### Degradation of sexual function genes

The results from both interspecific evolutionary analyses as well as intraspecific variation support the conclusion that genes responsible for sexual reproduction, specifically pollen function, have experienced disproportionate mutation accumulation. Domestication can increase the frequency of deleterious mutations, through both hitchhiking and the increased genetic drift of a bottlenecked population. Extended periods of clonal propagation can also reduce the ability to effectively purge deleterious mutations due to the lack of independent assortment and recombination (Muller 1964; Hill and Robertson 1966). Disentangling the relative contributions of domestication and clonal propagation is difficult in clonally propagated crops, as the 2 processes intertwine, and we will be unlikely to be able to fully attribute deleterious mutation accumulation to either process. Unlike other species like maize (Rodgers-Melnick *et al*. 2015) and humans (Hussin *et al*. 2015) where deleterious mutations are enriched in low recombination regions of the genome, in cassava, deleterious mutation abundance and recombination frequency show little correlation (Ramu *et al*. 2017). This suggests that the deleterious alleles that have reached fixation in cassava have done so on large haplotypic backgrounds, as expected with low recombination occurrence due to clonal reproduction. However, as recombination is not absent, cassava populations do not fit classical models of Hill–Robertson interference and Muller's ratchet (Felsenstein 1974; Charlesworth and Charlesworth 1997). Some cassava individuals maintain 2 divergent haplotypes, as may be expected with asexuality, but many do not (Qi *et al*. 2022).

Alongside this general accumulation of deleterious mutations, the disproportionate accumulation in genes related to sexual reproduction may be caused by relaxed selection on their function. Several domesticated clonal crop species have lost their sexual fitness (Mckey *et al*. 2010). One potential explanation of deceased sexual fitness is counter-selection against sex, where lower investment in sexual reproduction may allow for more energy toward yield-related traits as in potato (Simmonds 1997; Mckey *et al*. 2010). In cassava, selection against floral development may prevent branching, as branching precedes the development of flowers, but is an undesirable trait in agronomic contexts (Pineda *et al*. 2020). The decrease in population size from a domestication bottleneck can cause deleterious mutations to rise to high frequency (Bosse *et al*. 2019), and while domestication has also been linked to selection in flowering genes, especially in regard to photoperiod sensitivity (Lin *et al*. 2021), this may be less impactful for crop species such as cassava whose agronomic value is not derived from fruit or seed. The putative accelerated accumulation of mutations in sexually related genes may offer a snapshot of where in the genome these fitness impacts are experienced at higher rates.

### Conclusion

This work has produced a deep evolutionary resource for the evaluation of selection and deleterious mutations in cassava. Evolutionary conservation across the Euphorbiaceae family can help determine the functional importance of genes across the cassava genome. Since its domestication and transition to clonal propagation, cassava had an accumulation of deleterious mutations and a loss of overall fitness, especially regarding sexual reproductive fitness. Understanding the impacts of domestication and clonal propagation on genetic load in genes related to sexual reproduction can help overcome the reproductive hurdles in cassava breeding and may provide targets for gene editing to repair haplotypes otherwise beneficial to tuber traits necessary for cassava's economic value Fernandez-Pozo *et al*. 2015. These results address only one aspect of genetic load and deleterious mutations in cassava, but the evolutionary resource produced has the potential to address many more in the future. Understanding which genes are under selection pressure in cultivated cassava may help prioritize targeted genetic interventions for those genes that are likely functional and impactful. Genes responsible for sexual reproduction may serve as a target of breeding or genetic manipulation to maintain sexual fecundity for the continual breeding ability of cassava lines.

## Supplementary Material

jkae282_Supplementary_Data

## Data Availability

The inputs to analyses, code to reproduce tables and plots, and summary tables can all be found on the GitHub repository (https://github.com/em255/CassavaEuphorbiaceaeGeneEvolution). All sequence and assembly data used in this study were previously published (Long *et al*. 2023). Euphorbiaceae sequence reads and assemblies generated in this study are available under bioprojects PRJNA608937 on the Sequence Read Archives and PRJEB55682 on the European Nucleotide Archive, respectively. Supplemental material available at G3 online.
