# Supplementary material for: Evolutionary signatures of the erosion of sexual reproduction genes in domesticated cassava (Manihot esculenta)
Source: G3 (Bethesda). 2024 Dec 2;15(2):jkae282. doi: 10.1093/g3journal/jkae282 (PMC11797036; doi:10.1093/g3journal/jkae282)
Supplement: jkae282_Supplementary_Data [file jkae282_supplementary_data.zip › Supplemental_Figures_G3-2024-405407.pdf]

Supplemental Figures

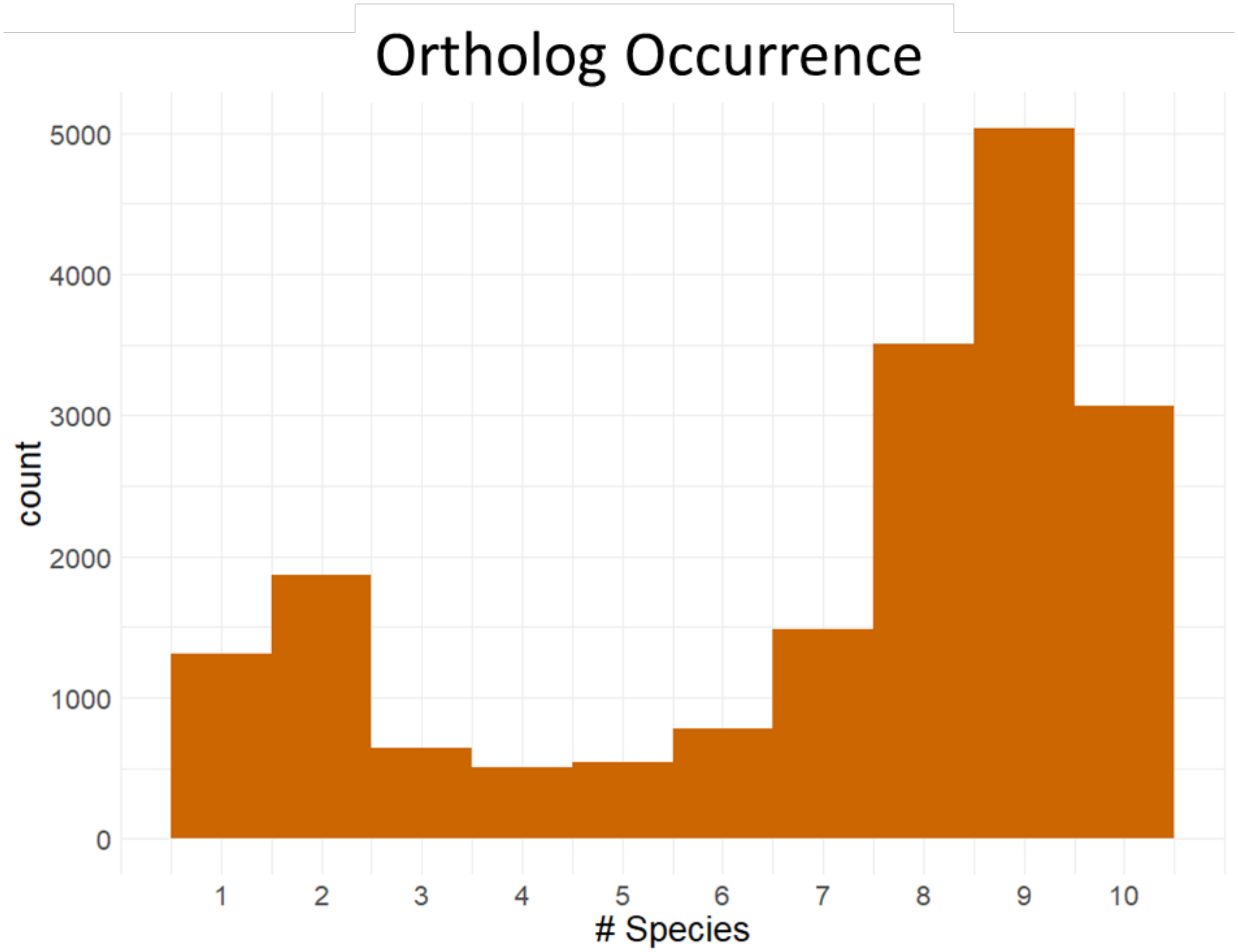

Supplemental Figure S1. Ortholog occurrence across high quality assemblies. An ortholog frequency histogram with the number of species, from among the 10 high quality genomes used in GENESPACE, that are represented in each ortholog group.

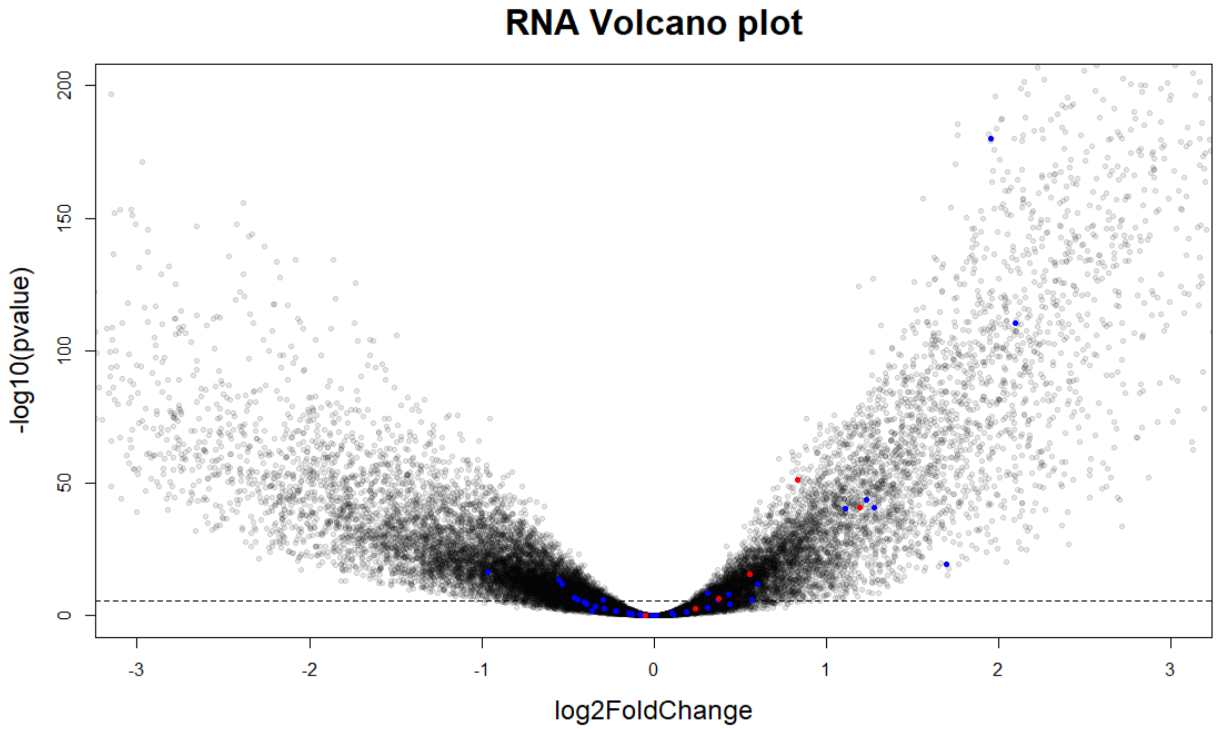

Supplemental Figure S2. Differential expression between flower and non-flower tissues. Log2Fold change of gene expression in flower tissues compared to non-flower tissues is shown (x-axis). From among the 48 relaxed genes (blue), 16 genes showed significant differential increased expression in flowers. From the 6 pollen related genes (red), 5 showed differential higher expression in flower tissues compared to non-flower tissues (blue).

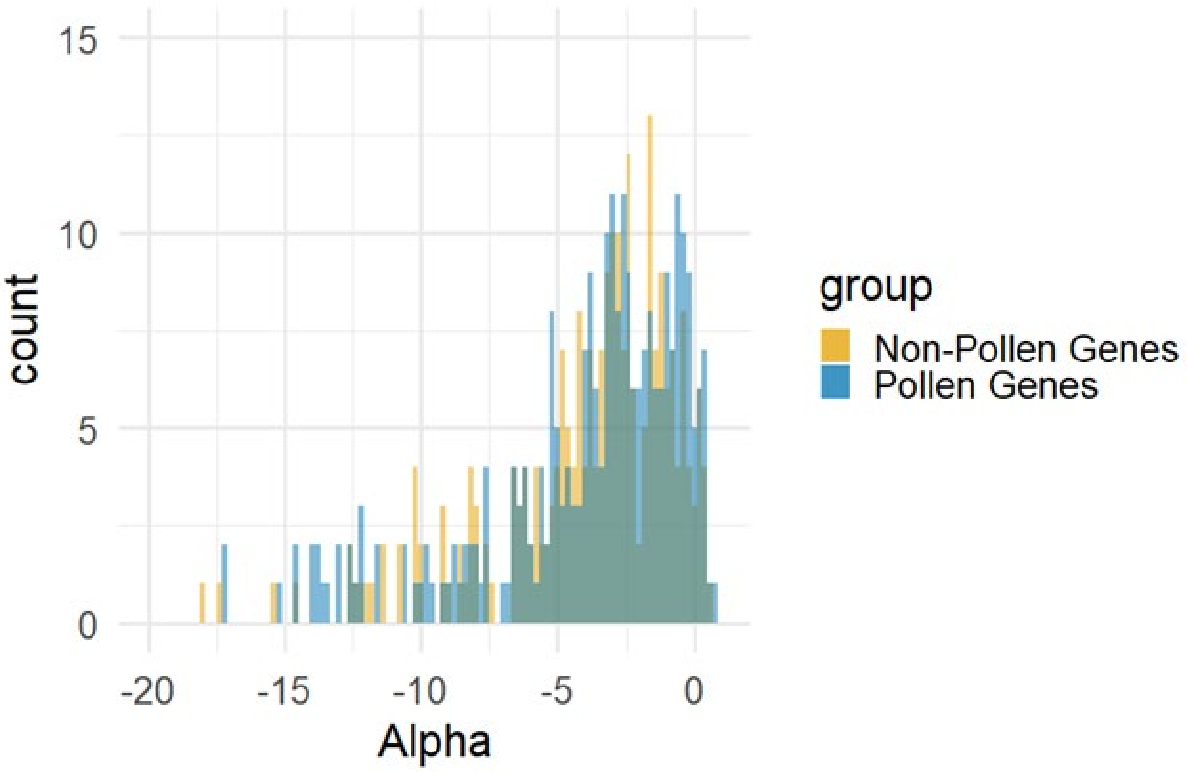

Supplemental Figure S3 Distribution of α values from MK test between pollen and non-pollen related genes. Histograms shown between pollen (blue) and non-pollen (orange) related genes for α. Non-pollen related genes are subsampled to an equal number of genes for visual comparison. No Significant difference between distributions was detected using a Chi-square test.

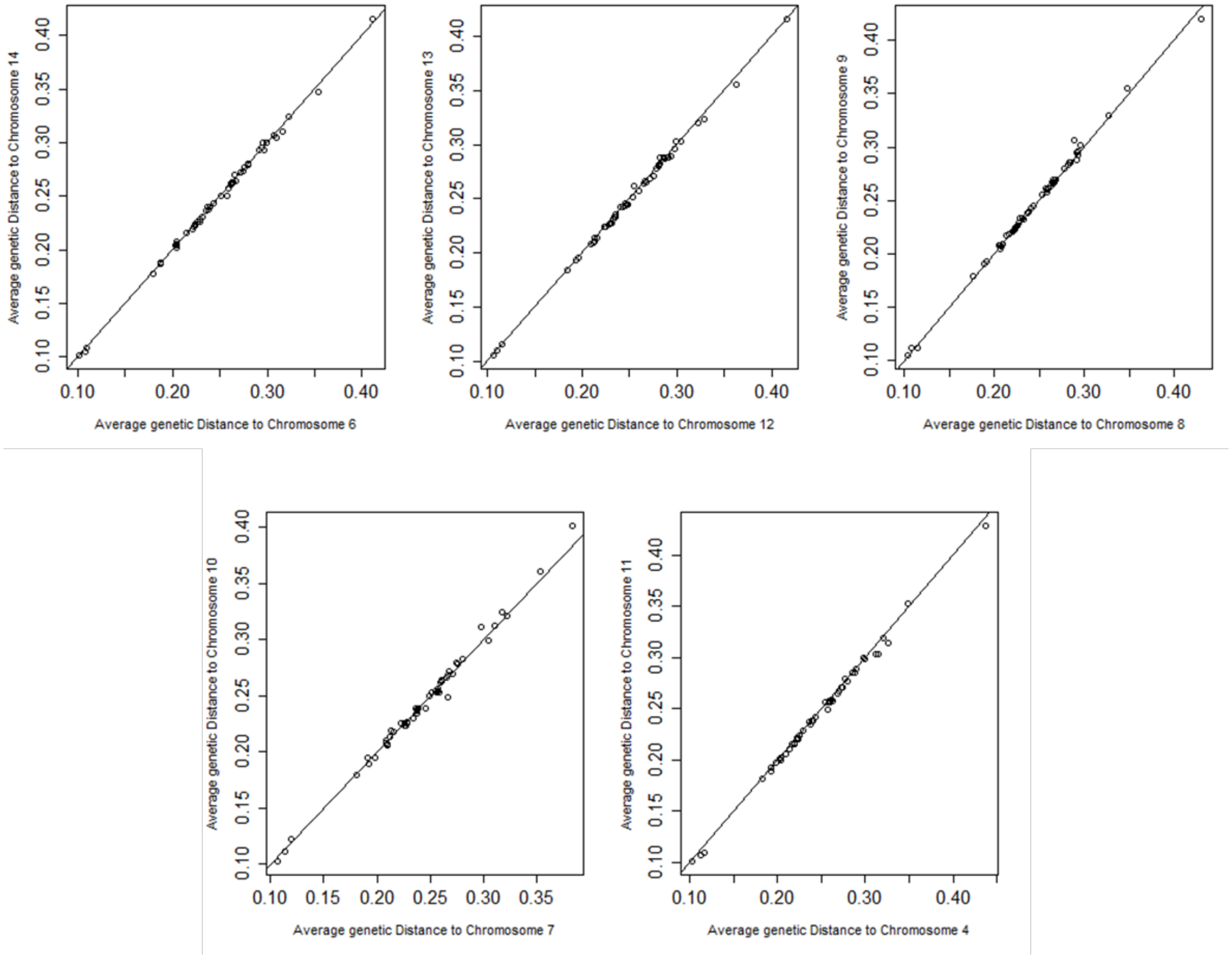

Supplemental Figure S4 Distances to each assembled genome between cassava homeologous averaged across all gens. chromosomes. Distances to each of the 52 assembled genomes were averaged over each orthologous gene. Distances were then plotted against each other, being paired by previously established ancestral homeologous chromosome pairs.

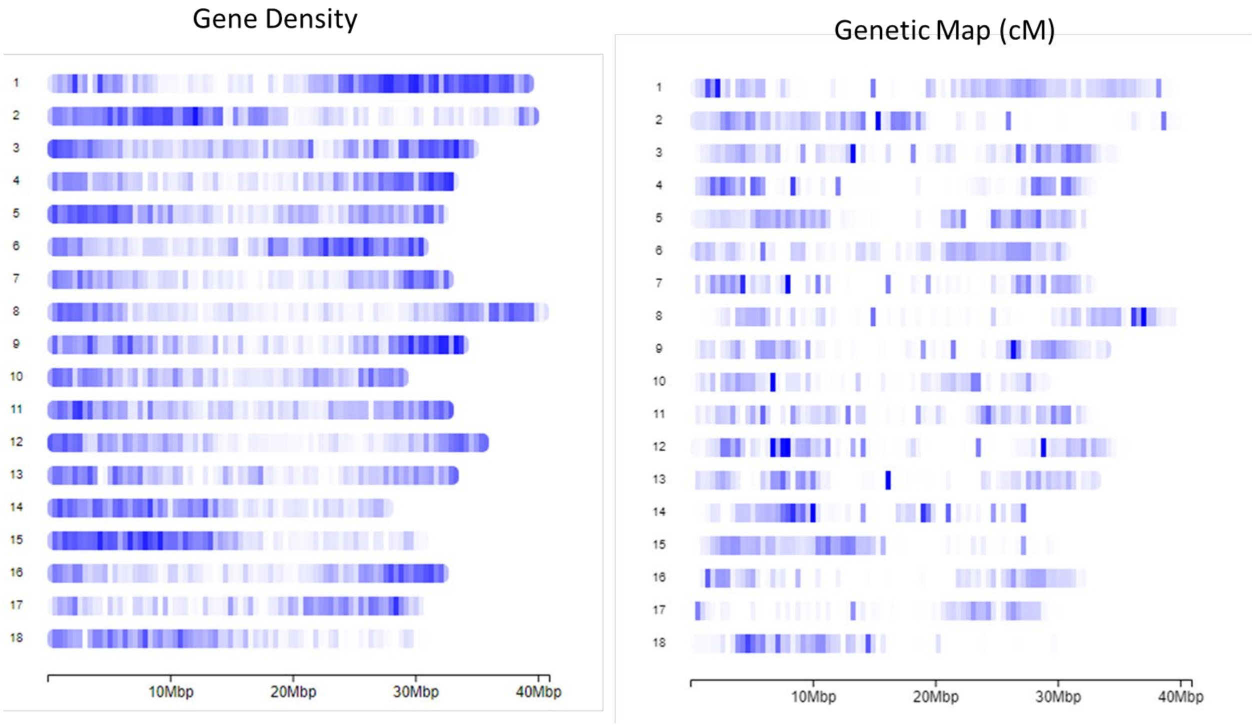
Gene Density
1
2
3
4
5
6
7
8
9
10
11
12
13
14
15
16
17
18
10Mbp
20Mbp
30Mbp
40Mbp
Genetic Map (cM)
1
2
3
4
5
6
7
8
9
10
11
12
13
14
15
16
17
18
10Mbp
20Mbp
30Mbp
40Mbp

23

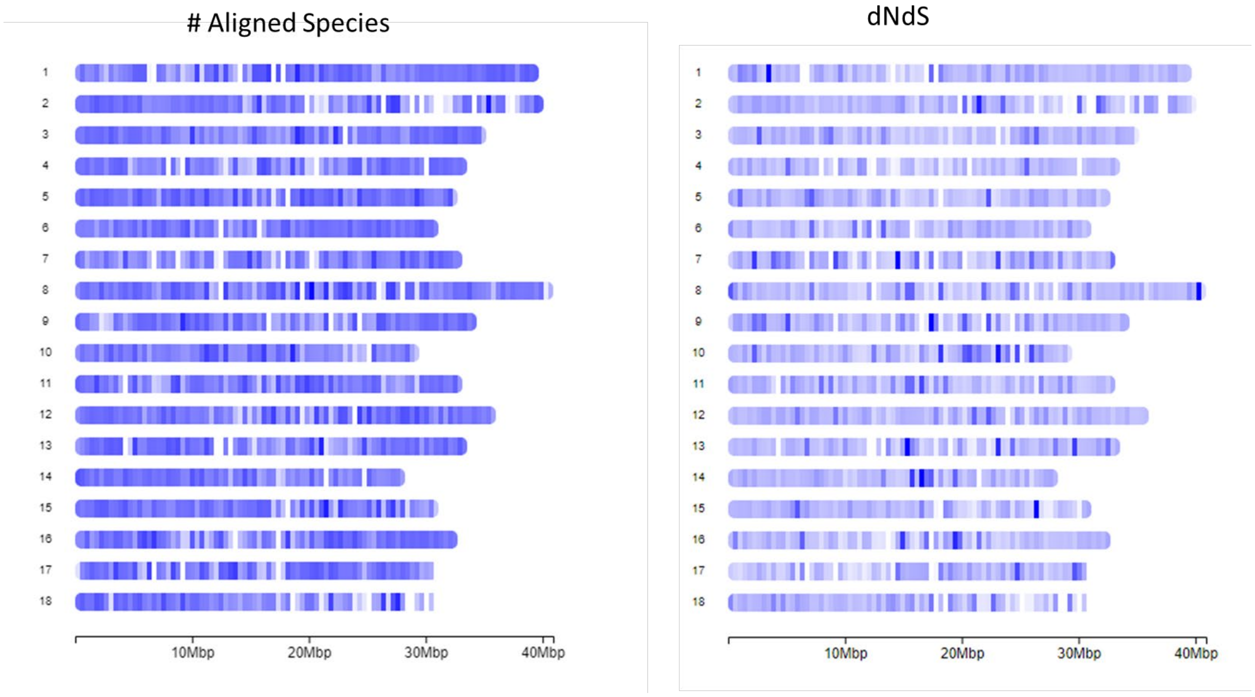
Aligned Species
1
2
3
4
5
6
7
8
9
10
11
12
13
14
15
16
17
18
10Mbp
20Mbp
30Mbp
40Mbp
dNdS
1
2
3
4
5
6
7
8
9
10
11
12
13
14
15
16
17
18
10Mbp
20Mbp
30Mbp
40Mbp

24

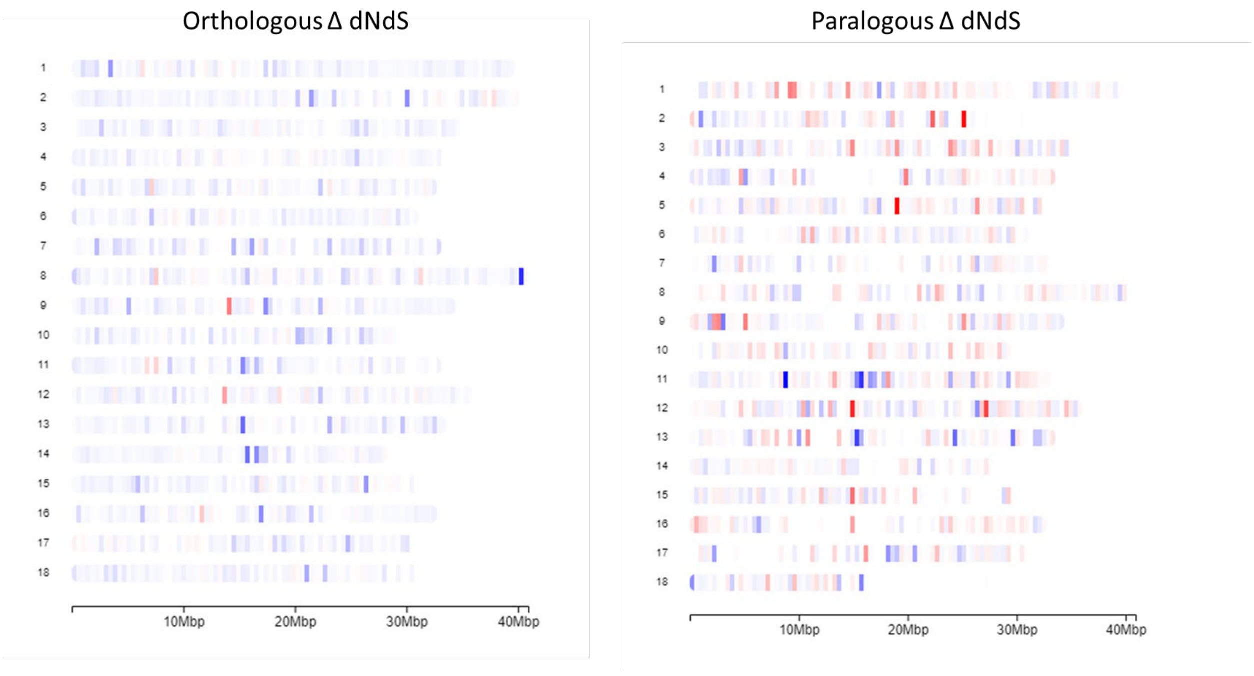
Orthologous Δ dNdS
Paralogous Δ dNdS
10Mbp
20Mbp
30Mbp
40Mbp

25

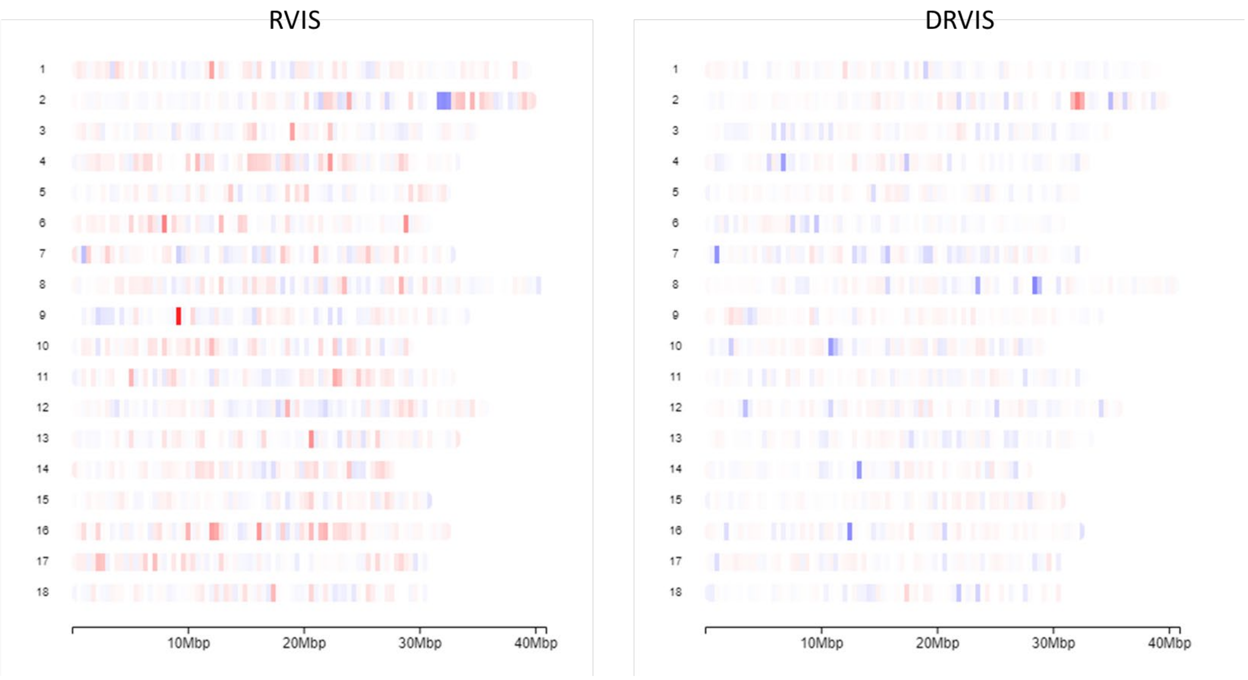
RVIS
DRVIS
10Mbp
20Mbp
30Mbp
40Mbp

26

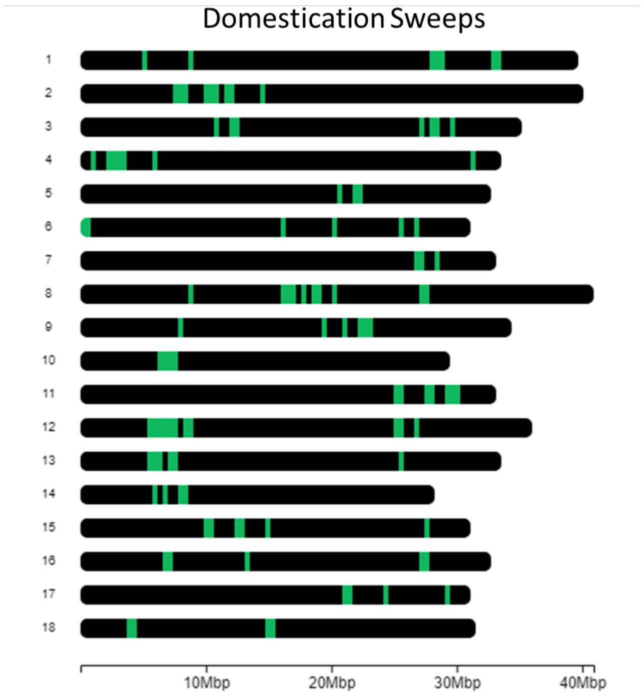

Supplemental Figure S5. Binned annotations across cassava chromosomes. Metrics including gene density, recombination rates (cM), dN/dS, number of aligned species, dN/dS differences between cassava and other species (Orthologous Δ dN/dS), dN/dS differences between gene copies (Paralogous Δ dN/dS), RVIS, DRVIS, and domestication sweeps were binned into 250kb windows for visualization.

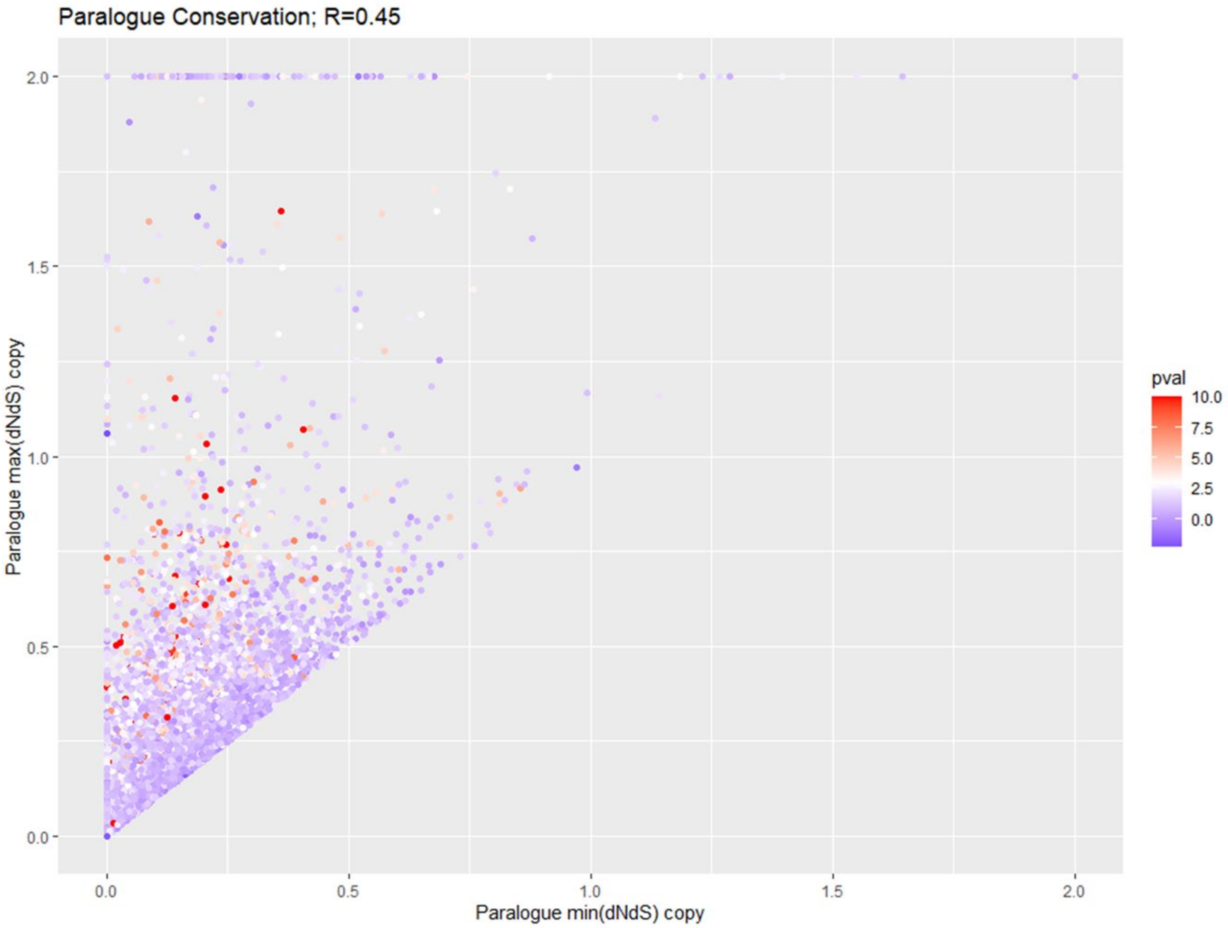

Supplemental Figure S6. Maximum and minimum values for paralogous gene copies. Among duplicated genes the dN/dS value for the most conserved copy (lowest dN/dS, x-axis) is compared to least conserved copy (highest dN/dS, y-axis). Correlation coefficient R<1 indicates asymmetrical conservation of paralogous gene copies. -log10(p-value) significance is indicated by color for differences between dN/dS in cassava and the Euphorbiaceae tree.
