# Supplementary material for: Evolutionary signatures of the erosion of sexual reproduction genes in domesticated cassava (Manihot esculenta)
Source: G3 (Bethesda). 2024 Dec 2;15(2):jkae282. doi: 10.1093/g3journal/jkae282 (PMC11797036; doi:10.1093/g3journal/jkae282)
Supplement: jkae282_Supplementary_Data [file jkae282_supplementary_data.zip › Supplemental_Tables_S2-S3_G3-2024-405407.pdf]

Supplemental Tables

Gene	Best-hit-arabi-name	BAR eplant - AtGenExpress eFP	AtGenExpress eFP - Sexual Reproduction Related	BAR eplant - Klepikova eFP	Klepikova eFP - Sexual Reproduction Related
Manes.01G017600	AT3G25500	NA	No	Axis of Inflorescence	Yes
Manes.01G224800	AT3G06720	Imbibed Seed	Yes	NA	No
Manes.01G257400	AT4G00500	senescent Leaf	No	senescent Leaf	No
Manes.02G048560	AT3G29090	Dry Seed	Yes	Dry Seed	Yes
Manes.02G134800	AT5G57655	Flower Petals	Yes	Silique 1	Yes
Manes.02G178800	AT1G76850	Cotyledons & Dry Seed	Yes	NA	No
Manes.02G207075	AT3G26935	Imbibed Seed	Yes	Stamen	Yes
Manes.02G213600	AT5G58520	2nd Internode	No	2nd Internode	No
Manes.02G218700	AT2G26640	Seed Stage 6	Yes	Root Apex	No
Manes.02G218800	AT2G26640	Seed Stage 6	Yes	Root Apex	No
Manes.02G222700	AT1G79600	Sepals	Yes	Silique 1	Yes
Manes.03G130950	AT2G32460	Mature Pollen	Yes	Anthers	Yes
Manes.03G167100	AT3G23160	Sepals	Yes	Root	No
Manes.03G182600	AT1G71830	Seed Stage 6	Yes	Flower	Yes
Manes.03G204900	AT3G04690	Mature Pollen	Yes	Anthers	Yes
Manes.04G009000	AT5G22640	Vegetative Rossette	No	Pedicel	Yes
Manes.04G017000	AT5G60740	Mature Pollen	Yes	Anthers	Yes
Manes.04G056400	AT1G03050	Mature Pollen	Yes	Anthers	Yes
Manes.04G084300	AT4G36220	senescent Leaf	No	senescent Leaf	No
Manes.04G095900	AT5G36880	Stamen	Yes	Stamen	Yes
Manes.04G153000	AT4G24480	Cauline Leaf	No	Cauline Leaf	No
Manes.04G165300	AT5G12380	Mature Pollen	Yes	Silique 5	Yes
Manes.05G004700	AT1G02550	Mature Pollen	Yes	Flower 1	Yes
Manes.05G041600	AT4G00230	Internode	No	Leaf Petiole	No
Manes.06G017100	AT2G02370	Dry Seed & Senescent Leaf	Yes	Senescent Leaf	No

Manes.06G064700	AT3G18030	Cotyledons & Dry Seed	Yes	Ovules	Yes
Manes.06G155400	AT5G24090	Stamens	Yes	Senescent Internode	No
Manes.08G037300	AT2G38910	Mature Pollen	Yes	Anthers	Yes
Manes.08G062900	AT3G56640	Mature Pollen	Yes	Flower 5	Yes
Manes.08G109400	AT1G56600	Dry Seed	Yes	Dry Seed	Yes
Manes.09G054600	AT1G57790	Leaf	No	Leaf	No
Manes.10G036100	AT2G01080	Dry Seed	Yes	Young Seed	Yes
Manes.10G068400	AT1G79610	Seed Stage 7	Yes	Silique 1	Yes
Manes.10G093300	AT5G64420	Imbibed Seed	Yes	Germinating Seed	Yes
Manes.11G075500	AT5G13930	Petals	Yes	Anthers	Yes
Manes.13G101500	AT2G22600	Mature Pollen	Yes	Stigmatic Tissue	Yes
Manes.13G121200	AT2G39980	Dry Seed and Flower Petals	Yes	Germinating Seed and Flower Petals	Yes
Manes.14G087750	AT5G60920	Leaf	No	Root Apex	No
Manes.14G149800	AT5G59720	Petals	Yes	Internode	No
Manes.15G073500	AT3G20530	Mature Pollen	Yes	Anthers	Yes
Manes.15G119800	AT2G13620	Mature Pollen	Yes	Anthers	Yes
Manes.15G139100	AT1G25240	Mature Pollen	Yes	Anthers	Yes
Manes.15G146200	AT5G42650	Cotyledons	No	Mature Leaf	No
Manes.16G101200	AT4G29750	Vegetative Rossette	No	Leaf	No
Manes.17G014100	AT2G35330	Seed Stage 7	Yes	Flower	Yes
Manes.18G019650	AT1G27680	Shoot Apex	No	Axis of Inflorescence	Yes
Manes.18G082700	AT1G77380	Anthers and Petals	Yes	Anthers	Yes
Manes.18G086900	AT1G77120	Cotyledons	No	Dry Seed	Yes

Table S2. Table of tissue specific expression of Arabidopsis thaliana homologs to significantly relaxed cassava.

GO.ID	Term	Annotated	Significant	Expected	P-Value
GO:0006412	translation	668	45	20.54	6.60E-08
GO:0000398	mRNA splicing, via spliceosome	241	22	7.41	3.70E-05
GO:0006511	ubiquitin-dependent protein catabolic pr...	564	36	17.34	7.40E-05
GO:0010344	seed oilbody biogenesis	13	4	0.4	0.00051
GO:0006890	retrograde vesicle-mediated transport, G...	47	7	1.45	0.00054
GO:0006893	Golgi to plasma membrane transport	27	5	0.83	0.00125
GO:0034762	regulation of transmembrane transport	60	6	1.84	0.00153
GO:0019915	lipid storage	30	5	0.92	0.00204
GO:0009860	pollen tube growth	177	15	5.44	0.00282
GO:0000278	mitotic cell cycle	351	12	10.79	0.00295

Table S3. Enriched GO Terms for MK- α Genes. GO term enrichment produced from "topGO" among genes in the top 5% of MK-α scores.
